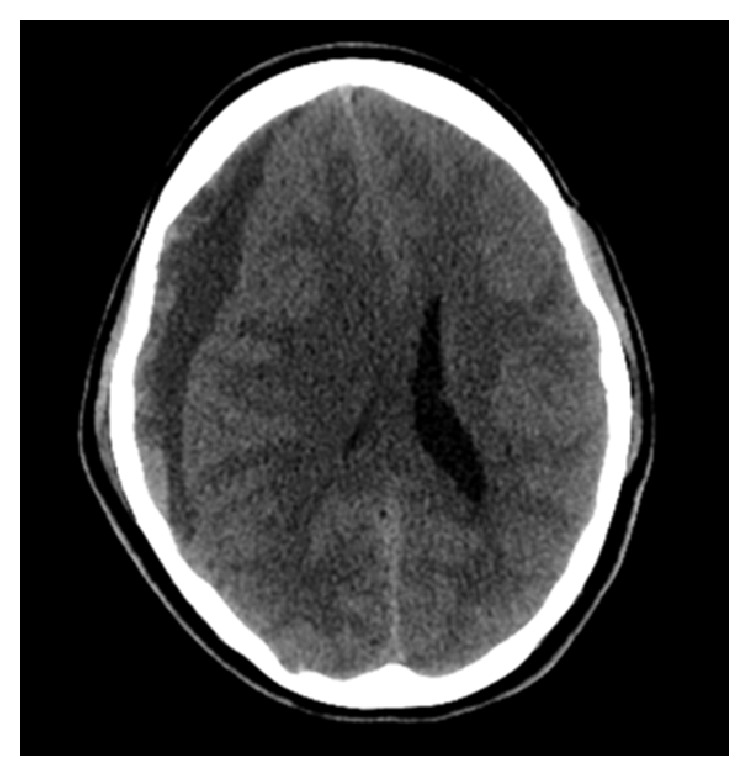# Subdural Hematoma as a Consequence of Epidural Anesthesia

**DOI:** 10.1155/2015/597942

**Published:** 2015-11-30

**Authors:** Tracy M. Bishop, Kareem S. Elsayed, Kathleen E. Kane

**Affiliations:** Department of Emergency Medicine, Lehigh Valley Hospital and Health Network/USF MCOM, CC & I-78, Allentown, PA 18103, USA

## Abstract

Regional spinal and epidural anesthesia are used commonly in operative procedures. While the most frequent complication, postdural puncture headache (PDPH), is a clinically diagnosed positional headache that is usually self-limited, subdural hemorrhage (SDH) is a potentially fatal complication that cannot be missed. We report a case of an otherwise healthy female who presented with persistent positional headache and was ultimately found to have a large subdural hematoma with midline shift requiring surgical evacuation.

## 1. Introduction

Spinal and epidural anesthesia are commonly used in regional anesthesia and are considered standard of care in obstetric anesthesiology. Complications occur in approximately 0.05% of cases [1]. The most common complication is a postdural puncture headache (PDPH); this is a clinical diagnosis that typically begins 24–48 hours following an inadvertent dural puncture and classically presents as a throbbing positional headache [2]. Most PDPH resolve within 5 days without intervention [2]. PDPH has been attributed to CSF leakage with a resultant loss in CSF pressure; subdural hematoma occurs due to rupture of vascular structures in the subdural space [2]. There are conflicting reports as to whether a blood patch in the setting of a potential dural puncture and clinically significant headache can prevent development of a subdural hemorrhage (SDH) [2, 3].

## 2. Case Report

A 33-year-old female presented to the Emergency Department (ED) with a chief complaint of headache. The headache began three weeks before, shortly after she completed a normal spontaneous vaginal delivery without complication. During labor, she had one unsuccessful attempt at placing an epidural catheter; the second attempt was successful. She reported that, for the past three weeks, the headache was constant but varied in intensity. During her pregnancy, she was diagnosed with gestational diabetes mellitus, which was managed conservatively. She denied history of chronic headaches.

Earlier, she had presented to another ED twice for treatment of the headache. Her primary care physician had diagnosed this headache as sinusitis and had prescribed her amoxicillin and pseudoephedrine. Her outpatient physician ordered an MRI to definitively diagnose the sinusitis; a radiologist read the MRI and asked her to go to an ED without discussing the results with her.

Upon arrival in the ED, she rated the headache as 9/10. The headache was worsened by moving her head and relieved when lying supine. She described mild photophobia. She denied blurred vision, nausea, numbness, weakness, or vomiting. Her review of systems was negative except for left ear pain for a week. She had sustained a very mild head injury one week before when she lost her balance and hit her forehead on the door of the bathroom while rising from the toilet. She denied loss of consciousness and stated that the intensity of the headache did not change. She denied smoking, alcohol, drug use, and recent travel. She denied family history of bleeding disorders. She adamantly denied domestic violence.

On examination, her vital signs were normal. She was awake, alert, and oriented ×3 with normal mood, affect, and speech. Her physical exam including a detailed neurological examination was normal.

Her laboratory tests revealed no coagulopathy. Computed tomography (CT) of the head was performed as the MRI was not immediately available. CT of the head (Figure 1) revealed a 2 cm right hemispheric subdural hematoma which appeared predominantly chronic but demonstrated superimposed acute or subacute hemorrhage. There was also subfalcine herniation to the left. No acute parenchymal hematoma or depressed skull fracture was identified.

The patient necessitated a neurosurgical consultation given the large right subdural hematoma with mass effect and left midline shift. A burr hole was placed in the operating room to drain the subdural hematoma. She also underwent a vertebral and carotid arteriogram that was negative for arteriovenous fistula and aneurysm. She received a blood patch and was discharged home with no neurological deficits. It is unlikely that the SDH resulted from her minor fall since her headache had been going on prior to that and was accompanied with ataxia. Therefore the minor fall was a sequel of an already existing subdural hematoma.

## 3. Discussion

Intracranial hemorrhage following spinal or epidural anesthesia for obstetric or other anesthetic reasons has been reported with both large and small gauge needles, with patients ranging from young adult to elderly [4]. In many cases, the SDH was found after the sudden onset of a very severe, debilitating headache, occasionally with rapid neurologic decompensation [5, 6]. However, Nepomuceno and Herd report a 17-year-old primigravida who suffered an inadvertent dural puncture during an attempt at epidural anesthesia. MRI of the head done four days after the attempt was normal; however, upon representation four weeks later, repeated MRI demonstrated bilateral subacute subdural hematomas [2].

It is prudent to consider intracranial hemorrhage in the postpartum patient with headache who underwent an epidural procedure. Many patients with postdural puncture SDH required surgical evacuation and delay in diagnosis may cause significant morbidity and mortality, especially in an often otherwise healthy population [4].

## Figures and Tables

**Figure 1:**